# A Private Quantum Bit String Commitment

**DOI:** 10.3390/e22030272

**Published:** 2020-02-27

**Authors:** Mariana Gama, Paulo Mateus, André Souto

**Affiliations:** 1 Instituto de Telecomunicações, 1049-001 Lisbon, Portugal; 2 Departamento de Matemática, IST, Universidade de Lisboa, 1049-001 Lisbon, Portugal; 3 LASIGE and Departamento de Informática, Faculdade de Ciências, Universidade de Lisboa, 1749-016 Lisboa, Portugal

**Keywords:** quantum bit commitment, privacy, entanglement, composable security, physical unclonable functions

## Abstract

We propose an entanglement-based quantum bit string commitment protocol whose composability is proven in the random oracle model. This protocol has the additional property of preserving the privacy of the committed message. Even though this property is not resilient against man-in-the-middle attacks, this threat can be circumvented by considering that the parties communicate through an authenticated channel. The protocol remains secure and private (but not composable) if we realize the random oracles as physical unclonable functions (PUFs) in the so-called bad PUF model.

## 1. Introduction

One of the most basic building blocks of complex cryptosystems is commitment schemes. A commitment scheme is a protocol that allows two mistrustful parties to interact in order to communicate some information that is set up a priori by the sender and that the receiver can only unveil at a later stage. In other words, it is just as if the message was sent inside a locked box, which can only be opened after the sender hands the key over to the receiver. The protocol is secure if the receiver cannot learn the message before the sender wishes to unveil it, and the sender cannot change the message after committing to it. Commitment schemes are used in several protocols, such as coin flipping, zero-knowledge proofs, and secure multiparty computation [1,2,3,4]. Since any weakness in the building blocks affects the security of the overall system, it is important to ensure that they are highly reliable.

Unfortunately, classical bit commitment (BC) schemes cannot be simultaneously unconditionally secure against a corrupted sender and a corrupted receiver, and Canetti and Fischlin proved that universally composable (UC) BC is impossible in the plain model [5]. Together with the impossibility proof, a UC commitment protocol in the common reference string model is provided in [5]. Similarly to the common reference string, the random oracle assumption also allows the existence of UC commitments [6,7].

In 1996, Lo and Chau [8] and independently Mayers [9] proved a no-go theorem for unconditionally secure quantum BC in the standard non-relativistic quantum cryptographic framework. Since then, many protocols relying on additional assumptions have been presented. Entanglement is one of the most extraordinary effects in quantum mechanics, and it is crucially important for quantum computing and quantum cryptography. There are multiple commitment schemes using EPR pairs, such as the one in [10], which is a purified analog of [11], and the relativistic and unconditionally secure protocols in [12] (note that, although secure commitment schemes can be obtained through the exploitation of relativistic constraints, these types of protocols are challenging to implement).

In this paper, we propose a new private commitment protocol, i.e., a commitment where the message is never announced, nor can it be derived from the messages exchanged between the parties. This property is attained through the use of entanglement. Since commitment protocols are mostly used as cryptographic primitives, it is of the utmost importance to study their security in different computational environments. As such, a strong emphasis is placed on the composability of these protocols. After characterizing the commitment functionality, the EPR pair trusted source functionality, and the random oracle functionality in Section 2, we show in Section 3 that these last two functionalities can be used as a resource to achieve a private commitment protocol with composable security, which is proven in Section 4. In Section 5, we analyze the security of the protocol in the bad PUF attack model. Section 6 features our final conclusions alongside with some directions for future work.

## 2. Preliminaries

A bit commitment protocol starts with the *commitment phase*, during which Alice chooses the value *m* she wants to commit to, and generates the pair (c,d). *c* is the *commitment*, which she immediately sends to Bob (who outputs a receipt message), and *d* is the *decommitment*, which she keeps to herself. In the *opening phase*, Alice sends (b,d) to Bob, who can either accept or reject. The protocol is said to be *concealing* if Bob cannot learn Alice’s committed message *m* before the opening phase, and *binding* if Alice cannot change her committed message *m* after the commitment phase.

**Figure 1:**
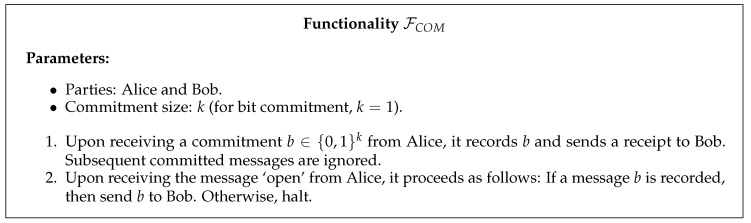
Commitment functionality.

The security of commitment protocols can be studied from a stand-alone perspective, with the requirements of concealingness and bindingness. However, since commitments are generally used as a subroutine of more complex tasks, it becomes mandatory for protocols to be secure in any computational environment. In a composable security proof, the parties running the protocol are considered as a single big party which must be indistinguishable from a simulated machine running an ideal functionality for commitment (see Figure 1).

**Figure 2:**
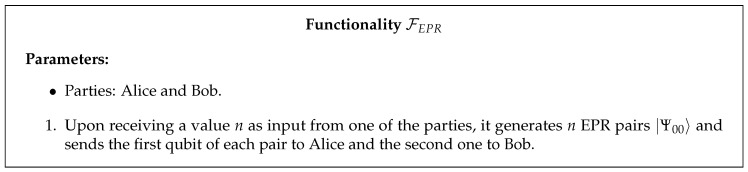
EPR pair source functionality.

In the protocol described in the next section, we assume that the parties have access to two different resources. The first one is an EPR pair trusted source modeled by the functionality in Figure 2. Note that the existence of this source is a very reasonable assumption since entanglement distribution has already been successfully implemented [13,14]. Before the beginning of the protocol, Alice and Bob can additionally sacrifice a small number of entangled pairs to estimate their correlation by using an algorithm such as the one described in Section 6.2 of [15]. Even if noisy quantum channels result in a loss of entanglement, the parties can run an entanglement distillation protocol and transform non-maximally entangled shared pairs into a smaller number of maximally entangled ones by using only local operations and classical communication (e.g., [16,17]—the last one is significantly less effective than the first, but has the advantage of being within the reach of current technology).

**Figure 3:**
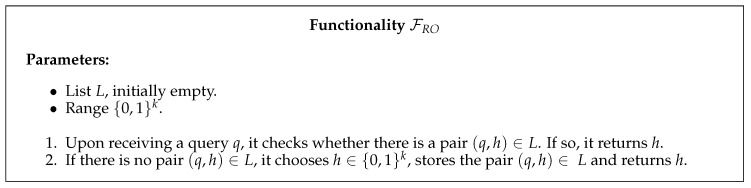
Random oracle functionality.

The second required resource, described by the functionality $F_{RO}$ in Figure 3, is named random oracle and behaves as an ideal cryptographic hash function, i.e., it maps each query to a fixed and uniformly random output in its range.

It is essential in our proof that a quantum computer cannot call the random oracle in superposition. Therefore, a realizable random oracle implementation cannot be a cryptographic hash function such as Secure Hash Algorithm (SHA).This fact makes the random oracle quite a strong assumption; nevertheless, it can be realized using physical unclonable functions (PUFs). PUFs are physical systems with some microscale structural disorder, which is assumed to be unique to each PUF and unclonable even by the PUF manufacturer. When external stimuli (challenges) are applied to a PUF, its response will depend on the disorder of the device. Therefore, each PUF *P* implements a unique function $f_{P}$ that gives responses $r=f_{P}\left(c\right)$ to challenges *c*. For more about PUFs, we refer to [18,19,20,21]. PUFs have a classical interface, and cannot be run in superposition, even by an all-powerful quantum adversary.

## 3. The Proposed Protocol

One of the characteristics of $F_{COM}$, the functionality for commitments, is that the message is never publicly announced. In most of the existing commitment protocols, nonetheless, the opening step includes sending the message over a public channel. Here, we propose a protocol (Protocol 1) that is not only composable, but also preserves the privacy of the message. We note that the privacy property is vulnerable to man-in-the-middle attacks: a third party, Eve, can pretend to be the EPR pair trusted source and send different sets of EPR pairs to Alice and Bob and then forward any received message. This can be prevented by adding an authenticated channel between Alice and Bob, as similarly done in quantum key distribution protocols.

The protocol will use as a resource the EPR pair trusted source functionality (Figure 2) and the random oracle functionality (Figure 3) presented in the previous section. It needs two instances of $F_{RO}$: $H_{1}$ with range ${\left\{0,1\right\}}^{2n}$ and $H_{2}$ with range ${\left\{0,1\right\}}^{n}$. Note that, unfortunately, we cannot use the weaker version of the RO, the global RO [7], since the programmability of the oracle is a key point of our security proof.

**Protocol 1** Private Quantum Bit String Commitment.**Message to be shared:**
$m=m_{1}\ldots m_{2n}$.**Setup:** Alice chooses a message size 2n and sends the value *n* to $F_{EPR}$. The functionality prepares the state $|\psi \rangle ={⨂}_{i=1}^{n}\;|\Psi_{00}\rangle$ and sends the odd qubits to Alice and the even ones to Bob.
**Commitment phase:**
1.To commit to a message *m*, Alice generates an uniformly random basis string $b\in {\left\{\left\{|0\rangle ,|1\rangle \right\},\left\{|+\rangle ,|-\rangle \right\}\right\}}^{n}$, where $|+\rangle =\frac{|0\rangle +|1\rangle }{\sqrt{2}}$ and $|-\rangle =\frac{|0\rangle -|1\rangle }{\sqrt{2}}$, and measures each of her qubits *i* in the basis $b_{i}$, obtaining outcomes $O\in {\left\{0,1\right\}}^{n}$. She then sends Bob the strings $c_{1}=m⊕H_{1}\left(b|O\right)$ and $c_{2}=H_{2}\left(b\right)$, where b|O is the concatenation of *b* and *O*.
**Opening phase:**
2.Alice sends the bases *b* to Bob.3.If $H_{2}\left(b\right)=c_{2}$, Bob accepts the opening, measures each of his qubits *i* in the basis $b_{i}$, obtaining outcomes $O^{\prime }\in {\left\{0,1\right\}}^{n}$, and calculates $m=c_{1}⊕H_{1}\left(b|O^{\prime }\right)$. Otherwise, he rejects.


## 4. Security Analysis

**Figure 4:**
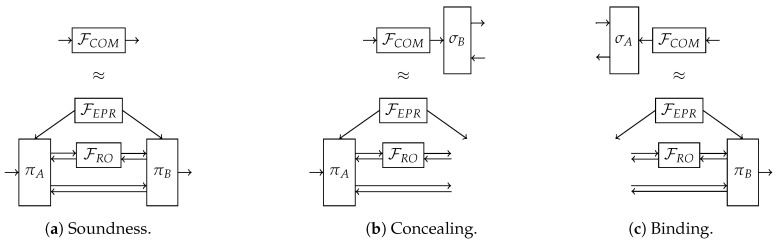
Conditions for the constructability of the resource $F_{COM}$ from the resources $F_{EPR}$ and $F_{RO}$ (**a**) corresponds to the soundness property by showing the equivalence between the ideal commitment functionality $F_{COM}$ and the protocol for honest parties (Alice and Bob behave according to $\pi_{A}$ and $\pi_{B}$, respectively); (**b**,**c**) correspond to security against dishonest Bob and Alice, respectively. Since the algorithm they follow is unknown, $\pi_{A}$ and $\pi_{B}$ are removed from the respective real system, while the simulators $\sigma_{A}$ and $\sigma_{B}$ are respectively added to the ideal system.

We proceed now to prove the security of Protocol 1 in the Abstract Cryptography framework [22] instantiated with quantum Turing machines [23]. The equivalences that need to be satisfied are depicted in Figure 4.

**Theorem** **1.** *Protocol 1 is composably secure. That is, the proposed commitment protocol constructs, from* $F_{EPR}$ *and* $F_{RO}$*, a resource that is within a negligible distance from the ideal resource* $F_{COM}$*, where simulators and distinguishers are modeled as quantum Turing machines.*

**Proof.**  This proof will be divided into three parts, one for each of the required equivalences. □

### 4.1. Soundness

Let |ψ〉 be the overall state of the system after Step 1. Note that

$$|\Psi_{00}\rangle =\frac{1}{\sqrt{2}}\left(|00\rangle +|11\rangle \right)=\frac{1}{\sqrt{2}}\left(|++\rangle +|--\rangle \right),$$

so, when Alice measures each of her qubits, the corresponding EPR pair will collapse to either |00〉 or |11〉 (for $b_{i}=\left\{|0\rangle ,|1\rangle \right\}$), or to either |++〉 or |−−〉 (for $b_{i}=\left\{|+\rangle ,|-\rangle \right\}$). Therefore, when Bob measures each of his qubits *i* in the basis $b_{i}^{\prime }=b_{i}$ he received from Alice in the opening phase, he will get exactly the same outcome as Alice, $O_{i}^{\prime }=O_{i}$, implying that $H_{1}\left(b^{\prime }|O^{\prime }\right)=H_{1}\left(b|O\right)$. Bob will then retrieve the message successfully, since $c_{1}⊕H_{1}\left(b^{\prime }|O^{\prime }\right)=m⊕H_{1}\left(b|O\right)⊕H_{1}\left(b^{\prime }|O^{\prime }\right)=m$.

### 4.2. Concealingness

Given any behavior of a dishonest receiver, we have to construct a simulator $\sigma_{B}$ that simulates $H_{1}$, $H_{2}$, and $F_{EPR}$ and provides the receiver with a commitment that can later be opened to the message in $F_{COM}$. Consider the following program for $\sigma_{B}$:

- *Simulation of $H_{1}$:* Whenever $\sigma_{B}$ receives the query b|O to $H_{1}$, it answers with $h=m⊕c_{1}$. In all other cases, it returns a value *h* as the ideal functionality would do and keeps (q,h) on a list of queries and respective answers.
- *Simulation of $H_{2}$:* Whenever $\sigma_{B}$ receives queries *q* to $H_{2}$, it returns a value *h* as the ideal functionality would do and keeps (q,h) on a list of queries and respective answers.
- *Simulation of $F_{EPR}$:* During the setup phase, $\sigma_{B}$ generates the state $|\psi \rangle ={⨂}_{i=1}^{n}\;|\Psi_{00}\rangle$, sends the even qubits to the corrupted receiver and keeps the odd ones to itself.
- During the commitment phase, upon receiving the receipt from $F_{COM}$, $\sigma_{B}$ chooses two uniformly random strings, $c_{1}\in {\left\{0,1\right\}}^{2n}$ and $b\in {\left\{\left\{|0\rangle ,|1\rangle \right\},\left\{|+\rangle ,|-\rangle \right\}\right\}}^{n}$, and measures each of its qubits *i* in the basis $b_{i}$, obtaining outcomes $O\in {\left\{0,1\right\}}^{n}$. It then sends $c_{1}$ and $c_{2}=H_{2}\left(b\right)$ to the corrupted receiver.
- During the opening phase, upon receiving the message *m* from $F_{COM}$, $\sigma_{B}$ sends the bases *b* to the corrupted receiver.

The behavior of $\sigma_{B}$ is the same regardless of the message that was sent to $F_{COM}$, and hence there is no algorithm for the dishonest receiver allowing him to guess the committed message with probability greater than $1/2^{2n}$.

### 4.3. Bindingness

Given any behavior of a dishonest sender, we have to construct a simulator $\sigma_{A}$ that simulates $H_{1}$, $H_{2}$, and $F_{EPR}$ and retrieves the message *m* from the sender’s commitment values and sends it to $F_{COM}$. It must also be able to detect when the sender is cheating and, whenever that happens, not send the opening message to $F_{COM}$. Consider the following program for $\sigma_{A}$:

- *Simulation of $H_{1}$ and $H_{2}$:* Whenever $\sigma_{A}$ receives queries *q* to $H_{1}$ or $H_{2}$, it returns a value *h* as the ideal functionality would do and keeps (q,h) on a list of queries and respective answers.
- *Simulation of $F_{EPR}$:* During the setup phase, $\sigma_{A}$ generates the state $|\psi \rangle ={⨂}_{i=1}^{n}\;|\Psi_{00}\rangle$, sends the odd qubits to the corrupted sender and keeps the even ones to itself.
- During the commitment phase, upon receiving the commitment strings $c_{1}$ and $c_{2}$ from the corrupted sender, $\sigma_{A}$ sends $m=c_{1}⊕H_{1}\left(b|O\right)$ to $F_{COM}$.
- During the opening phase, upon receiving the basis string $b^{\prime }$ from the corrupted sender, $\sigma_{A}$ sends the message ‘open’ to $F_{COM}$ if $b^{\prime }=b$. Otherwise, it does not open the commitment.

The real world receiver outputs error whenever the string $b^{\prime }$ sent by the sender is such that $H_{2}\left(b^{\prime }\right)\neq H_{2}\left(b\right)$. From the soundness property, we know that, when $b^{\prime }=b$, the receiver correctly retrieves the message. We are interested in the situation where $b^{\prime }\neq b$ (in which case the commitment will not be opened in the ideal world) and $H_{2}\left(b^{\prime }\right)=H_{2}\left(b\right)$. Since $F_{RO}$ is collision-resistant, this can only happen with negligible probability.

The addition of an authenticated communication channel makes this protocol a private and composable commitment protocol, which is yet to be achieved by classical cryptography based on the same assumptions.

## 5. Analysis in the Realistic Bad PUF Model

In order to study the security of PUF applications in a realistic scenario, the bad PUF attack model is described in [19]. In the bad PUF model, the fact that PUFs are real physical objects is exploited, and we consider both the simulatable bad PUFs, which possess a simulation algorithm that can be used by the manufacturer to compute responses to challenges and the challenge-logging bad PUFs, which allow the manufacturer to access a memory module in the device and read all the challenges applied to it (this malicious feature could also be added by an adversary after the construction of the PUF).

In our brief analysis, we consider that, in the proposed protocol (Protocol 1), the RO is replaced by PUFs. We may additionally suppose that the manufacturer (Alice, in our protocol), when in possession of a PUF, can program its responses to challenges. In this case, Alice should send $H_{1}$ to Bob at the end of the commitment phase, or else it would be easy for her to open a different message of her choosing without being caught. Protocol 2 describes a secure commitment in the bad PUF model where the adversary can program PUF responses. The requirement that the basis string *b* is a codeword of a minimum distance code will be important to guarantee security against a dishonest Alice. Note that, since the PUF responses may be programmed, $H_{2}$ can no longer be used by Bob to check the validity of the opening information and thus Protocol 2 only requires one PUF (represented by $H_{1}$). Instead, contrary to what happened in Protocol 1, Alice reveals the outcomes of her measurements in the opening phase. Bob then compares the revealed outcomes with his own measurement results in order to either accept or reject the opening. This does not affect the privacy of the protocol since only Bob has access to the PUF $H_{1}$ after the commitment phase.

**Protocol 2** Quantum Bit String Commitment with PUFs.**Message to be shared:**
$m=m_{1}\ldots m_{2n}$.**Setup:** Alice chooses a message size 2n and sends the value *n* to $F_{EPR}$. The functionality prepares the state $|\psi \rangle ={⨂}_{i=1}^{n}\;|\Psi_{00}\rangle$ and sends the odd qubits to Alice and the even ones to Bob. Alice prepares the PUF $H_{1}$.
**Commitment phase:**
1.To commit to a message *m*, Alice generates a uniformly random basis string $b\in {\left\{\left\{|0\rangle ,|1\rangle \right\},\left\{|+\rangle ,|-\rangle \right\}\right\}}^{n}$ such that *b* is a codeword of some pre-agreed code with minimum distance *d* and where $|+\rangle =\frac{|0\rangle +|1\rangle }{\sqrt{2}}$ and $|-\rangle =\frac{|0\rangle -|1\rangle }{\sqrt{2}}$. She measures each of her qubits *i* in the basis $b_{i}$, obtaining outcomes $O\in {\left\{0,1\right\}}^{n}$, and then sends Bob the PUF $H_{1}$ and the string $c_{1}=m⊕H_{1}\left(b|O\right)$, where b|O is the concatenation of *b* and *O*.
**Opening phase:**
2.Alice sends the bases *b* and the outcomes *O* to Bob.3.Bob measures each of his qubits *i* in the basis $b_{i}$, obtaining outcomes $O^{\prime }\in {\left\{0,1\right\}}^{n}$. If $O^{\prime }=O$, Bob accepts the opening. Otherwise, he rejects. He then calculates $m=c_{1}⊕H_{1}\left(b|O\right)$.

**Theorem** **2.** *Protocol 2 is unconditionally secure in the bad PUF model*.

**Proof.**  The soundness proof is similar to the one for Protocol 1. We now prove security against a dishonest Bob (receiver). □

### 5.1. Concealingness

Suppose that Bob wants to know the message *m* before the opening phase. After the commitment phase, he knows $c_{1}$ and is in possession of the PUF $H_{1}$. He might try to use $H_{1}$ and $c_{1}$ to get some information about the message. However, even if he knows $H_{1}$’s answer to every possible challenge, he still will not be able to get any information about the message from $c_{1}$, since every possible message will be equally likely.

Finally, we show that Protocol 2 is secure against a dishonest Alice (sender).

### 5.2. Bindingness

Suppose that Alice wants to change the committed message after the commitment phase. Before the opening phase, she has yet to send the basis string *b* and the measurement outcomes *O* to Bob. She might try to reveal a different basis string $b^{\prime }$ from what she used to measure her qubits. However, since $b^{\prime }$ must also be part of the same minimum distance code as *b*, Bob will end up measuring at least *d* of his qubits in the wrong basis. As was mentioned before, the outcomes $O_{i}^{\prime }$ of Bob’s measurements of these qubits will be uniformly random, and the probability of Alice revealing an outcome string $O^{\prime \prime }$ such that $O^{\prime \prime }=O^{\prime }$ is, therefore, $\frac{1}{2^{d}}$.

Classical commitments with PUFs have also been studied in the composability setting. In [20], PUFs were first formalized in the UC framework and an unconditionally secure commitment protocol was constructed. However, in this work, only honestly generated PUFs were considered and, in [21], a model where attackers can create malicious PUFs (very similar to the concept of bad PUFs) was proposed, together with a computational UC commitment scheme. Since then, it was shown in [24] that commitments with unconditional security can be obtained in the malicious PUF model and, in [25], an unconditional UC commitment in a stronger adversarial model (allowing PUF encapsulation) was presented. In these papers, it is assumed that, due to the nature of the PUFs, the simulator cannot simulate the answers of a PUF, and so it must honestly forward the queries to the PUF functionality. Protocol 2 is therefore clearly not composable since it is not equivocable, i.e., in the case of a dishonest Bob, $\sigma_{B}$ is unable to generate $c_{1}$ and $H_{1}$ during the commitment phase such that it can open it later to any message that happens to be in the functionality $F_{COM}$.

## 6. Conclusions

With this work, we achieved a commitment protocol that is not only composable but also private, since the message is never publicly announced. Man-in-the-middle attacks can be prevented by adding an authenticated channel. We suggest the use of physical unclonable functions to model random oracles, and note that the protocol remains secure and private (although not composable) if we consider the bad PUF attack model, which has been proven impossible for classical bit commitment without other assumptions. In future work, it would be important to obtain a protocol that remains composable in the bad PUF model, as well as analyzing the possibility of transmission errors or implementation-related vulnerabilities (as discussed in [26], for example).

Additionally, it is of interest to further study how to obtain composability in commitment schemes while using the minimum possible assumptions (for more on this topic, see [27]), and which of these assumptions are needed to achieve privacy.

## References

[1] Blum M. (1983). Coin Flipping by Telephone a Protocol for Solving Impossible Problems. ACM SIGACT News.

[2] Brassard G., Chaum D., Crépeau C. (1988). Minimum Disclosure Proofs of Knowledge. J. Comput. Syst. Sci..

[3] Damgård I., Fehr S., Lunemann C., Salvail L., Schaffner C. Improving the Security of Quantum Protocols via Commit-and-Open. Proceedings of the CRYPTO.

[4] Almeida Á.J., Loura R., Paunković N., Silva N.A., Muga N.J., Mateus P., André P.S., Pinto A.N. A brief review on quantum bit commitment. Proceedings of the SPIE, Volume 9286.

[5] Canetti R., Fischlin M., Kilian J. (2001). Universally Composable Commitments. Proceedings of the CRYPTO—Advances in Cryptology.

[6] Hofheinz D., Müller-Quade J., Naor M. (2004). Universally Composable Commitments Using Random Oracles. Theory of Cryptography.

[7] Canetti R., Jain A., Scafuro A. Practical UC Security with a Global Random Oracle. Proceedings of the 2014 ACM SIGSAC Conference on Computer and Communications Security.

[8] Lo H.K., Chau H.F. (1997). Is quantum bit commitment really possible?. Phys. Rev. Lett..

[9] Mayers D. (1997). Unconditionally secure quantum bit commitment is impossible. Phys. Rev. Lett..

[10] Kaniewski J., Tomamichel M., Hänggi E., Wehner S. (2013). Secure Bit Commitment From Relativistic Constraints. IEEE Trans. Inf. Theory.

[11] Kent A. (2012). Unconditionally secure bit commitment by transmitting measurement outcomes. Phys. Rev. Lett..

[12] Adlam E., Kent A. (2015). Deterministic relativistic quantum bit commitment. Int. J. Quantum Inf..

[13] Wengerowsky S., Joshi S.K., Steinlechner F., Zichi J.R., Dobrovolskiy S.M., van der Molen R., Los J.W.N., Zwiller V., Versteegh M.A.M., Mura A. (2019). Entanglement distribution over a 96-km-long submarine optical fiber. Proc. Natl. Acad. Sci. USA.

[14] Yin J., Cao Y., Li Y.H., Liao S.K., Zhang L., Ren J.G., Cai W.Q., Liu W.Y., Li B., Dai H. (2017). Satellite-based entanglement distribution over 1200 kilometers. Science.

[15] Renner R. (2005). Security of Quantum Key Distribution. Ph.D. Thesis.

[16] Bennett C.H., Brassard G., Popescu S., Schumacher B., Smolin J.A., Wootters W.K. (1996). Purification of Noisy Entanglement and Faithful Teleportation via Noisy Channels. Phys. Rev. Lett..

[17] Pan J.W., Simon C., Brukner Č., Zeilinger A. (2001). Entanglement purification for quantum communication. Nature.

[18] Pappu R., Recht B., Taylor J., Gershenfeld N. (2002). Physical One-Way Functions. Science.

[19] Rührmair U., van Dijk M. PUFs in Security Protocols: Attack Models and Security Evaluations. Proceedings of the 2013 IEEE Symposium on Security and Privacy.

[20] Brzuska C., Fischlin M., Schröder H., Katzenbeisser S. Physically Uncloneable Functions in the Universal Composition Framework. Proceedings of the CRYPTO 2011.

[21] Ostrovsky R., Scafuro A., Visconti I., Wadia A., Johansson T., Nguyen P.Q. (2013). Universally Composable Secure Computation with (Malicious) Physically Uncloneable Functions. Proceedings of the EUROCRYPT—Advances in Cryptology.

[22] Maurer U., Renner R. (2011). Abstract cryptography. Innovations in Computer Science.

[23] Mateus P., Sernadas A., Souto A. (2015). Universality of quantum Turing machines with deterministic control. J. Log. Comput..

[24] Damgård I., Scafuro A. Unconditionally Secure and Universally Composable Commitments from Physical Assumptions. Proceedings of the International Conference on the Theory and Application of Cryptology and Information Security.

[25] Badrinarayanan S., Khurana D., Ostrovsky R., Visconti I., Coron J.S., Nielsen J.B. (2017). Unconditional UC-Secure Computation with (Stronger-Malicious) PUFs. Proceedings of the EUROCRYPT—Advances in Cryptology.

[26] Pljonkin A. (2019). Vulnerability of the Synchronization Process in the Quantum Key Distribution System. Int. J. Cloud Appl. Comput..

[27] Lemus M., Yadav P., Mateus P., Paunković N., Souto A. On minimal assumptions to obtain a universally composable quantum bit commitment. Proceedings of the 2019 21st International Conference on Transparent Optical Networks (ICTON).

